# Mechanics of Bio-Inspired Protective Scales

**DOI:** 10.3390/biomimetics10020075

**Published:** 2025-01-25

**Authors:** Antonio Pantano, Vincenzo Baiamonte

**Affiliations:** Dipartimento di Ingegneria, Università degli Studi di Palermo, 90128 Palermo, Italy; vicio.baiamonte@hotmail.it

**Keywords:** scales, protective systems, puncture-resistant, biomimetics

## Abstract

Natural armors found in animals like fish and armadillos offer inspiration for designing protective systems that balance puncture resistance and flexibility. Although segmented armors have been used historically, modern applications are hindered by a limited understanding of their mechanics. This study addresses these challenges by presenting two novel bio-inspired scale structures with overlapping and staggered configurations, modeled after the elasmoid designs found in fish. Their shapes differ significantly from other artificial scales commonly described in the literature, which are typically flat. Instead, these scales feature a support that extends vertically from the substrate, transitioning into an inclined surface that serves as the protective component. Finite element method tests evaluated their performance in puncture resistance and flexibility. The results showed that one type of scale provided better puncture resistance, while the other type offered greater flexibility. These findings highlight how small geometric variations can significantly influence the balance between protection and flexibility. The results offer new insights into the mechanisms of natural armor and propose innovative designs for personal protective equipment, such as bulletproof vests, protective gloves, and fireproof systems. The finite element simulations employed to test the protective systems can also serve as valuable tools for the scientific community to assess and refine designs.

## 1. Introduction

There are multiple examples of animals equipped with protective systems. In any type of natural application, the protective structures are mainly used to defend the epidermis of the animal in question from the attack of other animals and from natural environmental adversities that it is facing. In reality, often, these dermal protective systems also have secondary functions, aside from simple protection of the body. In fact, they are multifunctional, ensuring, for example, reduction in hydrodynamic friction in fish, coloring by camouflage in some reptiles, luminescence, and regulation of temperature. Flexible natural armors are attracting increasing attention for their unique combinations of hardness, flexibility, and lightness. The extreme contrast of the rigidity between hard scales and surrounding soft tissues inspires the design of biomimetic protection systems that are simultaneously difficult to drill and at the same time flexible. In particular, it has been seen that fish have protective structures formed by small overlapping and staggered small elements, scales, which accentuate the protective effects, without influencing in any way the weight and the ability to move. Thus, in the biomimetic field, research was carried out to create protective solutions inspired by the hierarchical structures in scales seen in nature [1,2,3,4,5,6,7,8,9,10,11,12,13,14,15,16,17,18,19,20,21,22,23,24,25,26,27,28,29,30,31,32,33,34,35,36,37,38,39,40,41]. It is believed that these can be applied by humans to improve the protective systems currently in use, such as gloves, bulletproof vests, or other.

A review of flexible dermal armor for the protection of fish, reptiles, and mammals has been delivered by Yang et al. [1]. Another review by Naleway et al. [2] presents the basic building blocks of the structural biological materials and a variety of protective strategies in marine organisms, with a focus on their structure and mechanical properties and a discussion on the bio-inspired potential of these biological materials. Natural protective systems are distinguished by their hierarchical structures, which integrate hard and soft components to optimize mechanical performance. Fish scales, for example, exhibit overlapping designs with mineralized layers and collagen fibers that provide both flexibility and puncture resistance [1,2,3,4,5,6,7,8,20,30]. Ganoid scales, such as those of alligator gar, demonstrate unique anisotropic properties due to their ganoine layer and bony foundation [3,4,19]. The elasmoid scales of teleost fish, like carp, feature Bouligand structures that enhance toughness and energy dissipation [3,4,20]. Marine organisms such as chitons and seahorses also display remarkable protective adaptations. Chiton girdle scales offer flexibility and protection through their tessellated arrangement [36], while the articulated armor of seahorses balances flexibility and resistance to crushing [22]. Shark skin, characterized by denticles, combines hydrodynamic efficiency with mechanical resilience [29]. Osteoderms in reptiles, such as alligators and armadillos, further highlight the role of mineralized structures in providing mechanical support and thermal regulation [15,16]. Pangolin and turtle scales offer insights into hierarchical design principles where overlapping structures enhance both flexibility and damage resistance [17,18].

The mechanical properties of natural protective systems are as diverse as their structures. Studies on pangolin scales reveal their ability to resist deformation through overlapping keratinous plates [17,39]. Fish scales, such as those of striped bass and arapaima, demonstrate nonlinear flexural behavior that supports locomotion and energy absorption [27,28]. Armadillo and turtle shells, composed of osteoderms, achieve toughness and puncture resistance through their hierarchical and mineralized structures [16,18]. The dynamic response of fish scales under high strain rates has also been explored, revealing enhanced puncture resistance due to the layered microstructure [21]. Seahorse armor, with its segmented bony plates, provides insights into fracture resistance and prehensile capabilities [27]. Similarly, the mechanics of striped bass skin have been linked to the synergistic behavior of scales and underlying collagen layers, which act as an exotendon during locomotion [28]. Studies on the mechanical response of biomimetic materials, inspired by these systems, highlight the potential to replicate their toughness and flexibility in synthetic designs [10,27]. The balance between hardness and compliance observed in these systems underpins their ability to provide protection without compromising mobility, a principle that has been extensively applied in bio-inspired materials research.

Advanced computational approaches have been instrumental in understanding the mechanics of natural protective systems. Finite element models have been used to simulate the deformation of fish scales and segmented armors, revealing the effects of geometry and material properties on their mechanical behavior [8,31,32]. Discrete element methods have further elucidated the interaction between individual scales and soft substrates, providing insights into their anisotropic response [27,32]. Frictional effects in biomimetic scales have been analyzed to understand their role in nonlinear mechanical behavior, while coupled bend–twist mechanics have demonstrated the complex kinematics of scale systems [34,35]. Analytical models, combined with experimental data, have helped predict the dynamic response of fish scales under various loading conditions, offering guidelines for optimizing bio-inspired designs [8,14]. Further computational studies have delved into the role of scale morphology and interlocking patterns on puncture resistance and energy absorption. For instance, models of elasmoid fish scales have shown how interscale friction and overlapping arrangements enhance mechanical performance under dynamic loading conditions [13,26]. These insights provide a foundation for the design of advanced flexible composites and protective materials.

The translation of natural design principles into synthetic materials has led to groundbreaking advancements in bio-inspired armor and flexible composites. Three-dimensional printing has enabled the fabrication of bio-inspired structures, replicating the overlapping and hierarchical arrangements of fish scales to enhance mechanical performance [14,40]. Segmented ceramic armors, inspired by osteoderms and fish scales, have been developed to balance toughness and flexibility in protective applications [4,39]. Biomimetic shark skin and fish scales have found applications in hydrodynamics and energy absorption, demonstrating the versatility of bio-inspired designs [13,29]. The unique morphology and mechanical adaptability of these systems have also inspired innovations in robotics, aerospace, and wearable technologies [25,26]. Additionally, studies on pangolin and armadillo scales have led to the development of materials with enhanced resistance to penetration and impact, providing insights into multifunctional armor systems [15,17].

**Figure 1:**
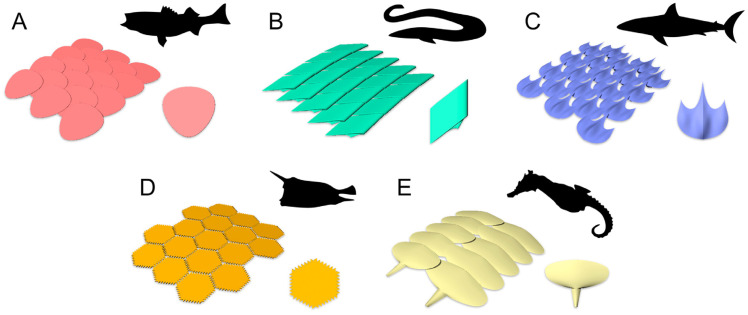
Representative schematics of common protective armors among living fishes. (**A**) Overlapping elasmoid scales common among most teleosts, such as striped bass; (**B**) interlocking ganoid scales common among gars and bichirs; (**C**) partially imbricated placoid scales common among sharks; (**D**) tessellating carapace scutes common among boxfishes; (**E**) interlocking and overlapping bony plates common among seahorses and related syngnathid fishes. Reproduced with permission from M. M. Porter et al., J. of the Mech. Behavior of Biomedical Mat., 73 (2017) 114–126. ©2017, Elsevier [3].

**Figure 2:**
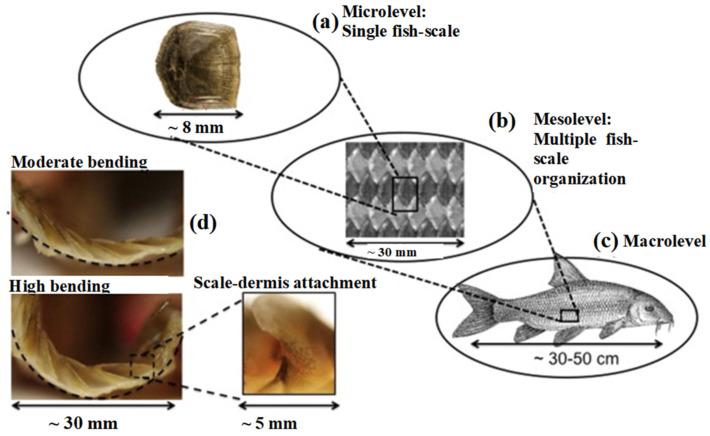
Structure of fish skin from the microlevel (single fish–scale) (**a**), to the mesolevel (**b**), to the macro-level (full skin) (**c**). Bottom left figures, (**d**), Subfigures (**d**) illustrate the deformation of fish skin during bending, highlighting a pronounced rotation of individual scales. Reproduced with permission from F. J. Vernerey et al., Int. J. of Solids and Structures, 51 (2014) 274–283. ©2017, Elsevier [8].

Fish have protective systems designed to protect them both from attacks by other fish, or predators and to reduce resistance to hydrodynamic advancement. These systems are formed by hierarchical structures comprising scales of resistant materials placed on a soft substrate, that is, the skin of the fish. The scale systems present in fish vary both according to the characteristics of the individual scales in terms of shape, size, and material and according to their arrangement on the soft substrate. Fish have protective structures with overlapping scales; however, the degree of overlap is a variable factor in the transition from one species to another. Among the main factors that influence the behavior of the coating in terms of protective capacity and motion flexibility, there is the type of scales a fish has. There are four types of scales, namely placoid, ganoid, cosmoid, and elasmoid (which have two sub-categories, ctenoid and cycloid). Figure 1 and Figure 2 show several types of scales, also presenting the typical degree of overlap that can be found with fish having this type of scale.

In recent years, strong research has been carried out on protective scale structures to identify possible biomimetic solutions inspired by fish. In particular, synthetic models were created, reproducing the hierarchical structure in scales, and mechanical tests were subsequently performed. A review of recent studies that use 3D printing techniques to investigate the biomechanics of armored fishes specifically was provided by Porter et al. [3]. To understand the behavior of the structure, these tests were performed with the variation of some characterizing parameters, such as scale material, material of the substrate representative of the skin, scale geometry, size of the scales, and degree and type of overlap of the scale structure. Martini et al. [4] used 3D printing to fabricate arrays of scales with increasingly complex geometries and arrangements, from simple squares with no overlap to complex ganoid-scales with overlaps and interlocking features. Stiff polymer (ABS) scales were glued onto a much softer substrate (polyurethane) with a thickness of 3 mm; then, they performed puncture tests and flexural tests on each of the 3D-printed materials and reported the puncture resistance and compliance characteristics of each design. In another study, Martini et al. [5] studied flexible bio-inspired armor based on overlapping ceramic scales, high purity alumina, and a soft silicone elastomer for the membrane. The fabrication combined laser engraving and a stretch-and-release method, which allowed for fine tuning of the size and overlap of the scales; they found out that compared to a continuous layer of uniform ceramic, the fish-scale like armor is not only more flexible but also more resistant to puncture and more damage-tolerant. The proposed armor is also about ten times more puncture-resistant than soft elastomers, making it a very attractive alternative to traditional protective equipment.

Some experiments were performed also directly on the fishes. Szewciw et al. [6] investigated the flexural response of whole teleost fish and how scales affect global flexural stiffness. A bending moment was imposed on the entire body of a striped bass (*Morone saxatilis*). Imaging was used to measure local curvature; it was found that the flexural stiffness is the highest in the thick middle portion of the fish and lowest in the caudal and rostral ends. The flexural response is nonlinear, with an initial soft response followed by significant stiffening at larger flexural deformations. In another work, Szewciw et al. [7] explored the possible additional role of the skin in fish undulatory locomotion by examining the structural and mechanical properties of the dermal stratum compactum layer of striped bass (*Morone saxatilis*) skin. The structure, mechanical response, and function of stratum compactum was investigated by combining several methods: optical microscopy and histology, tensile tests on descaled skin specimens in different anatomical locations and orientations, puncture tests, and flexural tests on whole fish with disruption of the s. compactum.

**Figure 3:**
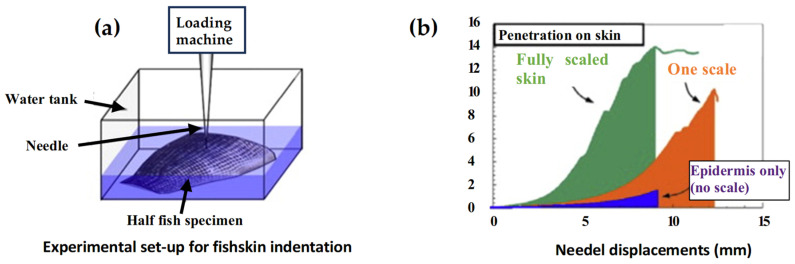
Illustration of the indentation test performed on fish skin and their results: (**a**) experimental set-up for the indentation, (**b**) graph of the force-deflection curve. Reproduced with permission from F. J. Vernerey et al., Int. J. of Solids and Structures, 51 (2014) 274–283. ©2017, Elsevier [8].

To obtain a better understanding of the scale mechanics, the finite element method (FEM) was used to perform parametric studies. Vernerey et al. [8] presented a computational methodology to analyze the mechanical behavior of thin composite structures with a direct application to fish skin. The proposed procedure was applied to the specific case of the fish skin structure of the *Morone saxatilis*, using a computational finite element approach. The numerical study shows that fish skin possesses a highly anisotropic response, with a softer bending stiffness in the longitudinal direction of the fish. This softer response arises from significant scale rotations during bending, which induce a stiffening of the response under large bending curvature. In Figure 3, the indentation test and results performed on the fish skin by Vernerey et al. [8] is reported. The results show that the presence of scales increases the penetration resistance of the skin by almost tenfold; moreover, scale interlocking further improves the performance of the protective system.

**Figure 4:**
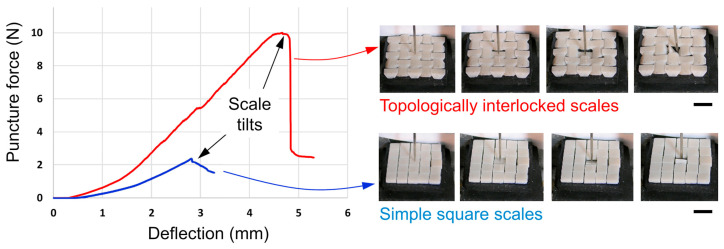
Puncture force–deflection curves for simple and interlocked scales with associated sequences of pictures. Both systems fail by sudden tilting of the indented scale. However, tilting is delayed in the interlocked scales, which increases the puncture resistance by a factor of four. Scale bars: 10 mm. Reproduced with permission from M. M. Porter et al., J. of the Mech. Behavior of Biomedical Mat., 73 (2017) 114–126. ©2017, Elsevier [3].

The effect of the interactions between neighboring scales is very important, as it was proved by the work of several authors (i.e., [3,4,8]). A scale resting on a softer substrate when indented by a sharp needle because of the relatively high strength of the scales and the high compliance of the substrate fails by tilting. A topologically interlocked array of scales, which is what is commonly found in nature, when subjected to puncture delays the tilting of the indented scale and can increase the puncture resistance by a factor of four. Figure 4 highlights the improvement in puncture resistance for the topologically interlocked array of scales.

Recently, Dura et al. [39,40] examined how the shape of scales affects the mechanical performance of bio-inspired protective structures. Using experiments and simulations, the researchers found that specific scale geometries optimize the balance between flexibility and stiffness [39]. In [40], Dura et al. investigated the energy absorption of 3D-printed composite structures inspired by fish scales. Low-velocity impact tests showed that the bio-inspired design significantly enhances energy absorption, making these structures suitable for impact-resistant applications. Xuechen et al. [41] explored the role of pangolin scales in pathogen defense. Using multi-omics analysis, the researchers found that the scales trap pathogens and contain antimicrobial proteins and metabolites, suggesting a complex defense system that enhances innate immunity.

Protection and flexibility are aspects that collide with each other, but the creation of protective scale structures allows for greater design possibilities to optimize both aspects as needed. For example, Martini et al. [4] in their study show that the simplified ganoid and elasmoid designs, which are close to the geometry and arrangement of the natural scales, are among the most efficient designs, providing the highest average resistance to perforation and without losing the maximum bending compliance, as compared to other designs that cover the entire surface. Natural evolution has shaped the geometry and arrangement of natural scales to maximize protective efficiency.

This study examines the puncture resistance and flexibility of natural dermal armor, offering insights into its design principles and potential applications in bio-inspired protective systems. Advances in additive manufacturing and computational modelling can boost the design, the optimization, and the production of protective structures inspired by fish scales for various applications. Here, two novel bio-inspired scale structures are presented that are based on the elasmoid designs present in fishes. Their shapes differ significantly from other artificial scales commonly described in the literature, which are typically flat. Instead, these scales feature a support that extends vertically from the substrate, transitioning into an inclined surface that serves as the protective component. Their performances are analyzed by finite element simulation. In particular, the two structures were tested both with regard to the guaranteed protection, through perforation tests, and the flexibility allowed, through flexural tests. The nonlinear flexural response is characterized by low stiffness at small curvatures due to scale sliding and significant stiffening at larger curvatures as they engage in protection. These results can guide the development of advanced, efficient protective materials inspired by natural armors.

## 2. Materials and Methods

### 2.1. Novel Scale Models

The two novel shapes proposed stand out distinctly from the flat artificial scales commonly described in the literature. These scales incorporate a vertical support extending from the substrate, transitioning into an inclined surface that functions as the protective component. The design aims to increase the distance between the top of the scale and the substrate, requiring greater deformation of the scales when subjected to pressure. This enhanced deformation allows the scales to absorb more energy, thereby improving the substrate’s ability to withstand loads. In contrast, the traditional flat scale design merely provides a hard surface to shield the softer substrate and redistributes pressure over a larger area.

The spacing of the scales on the substrate has been carefully designed to allow for slight overlap at the edges, ensuring adequate protection while preserving the overall structure’s lightness and flexibility. The thickness of the scales has been carefully selected to ensure they are robust enough to withstand loads by deforming without breaking, while still maintaining a lightweight profile.

#### 2.1.1. Type 1 Bio-Inspired Scale

**Figure 5:**
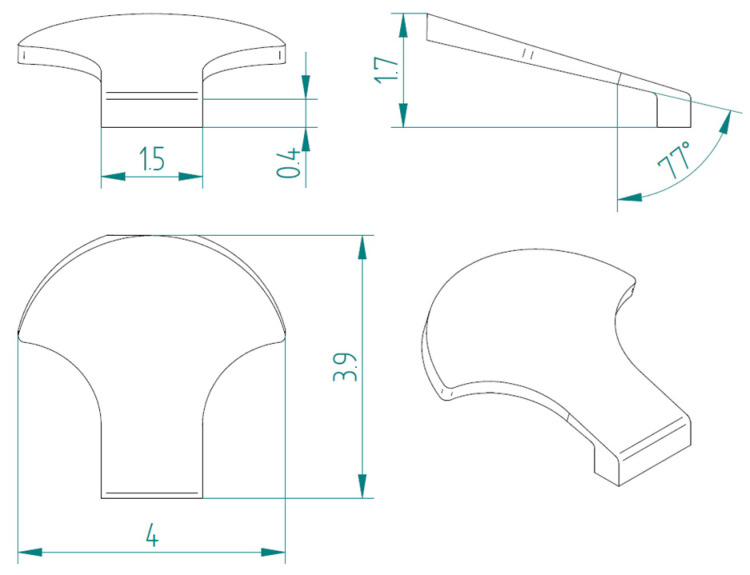
Type 1 bio-inspired scale with geometric dimensions in millimeters.

The first type, referred to as type 1, of bio-inspired scale is shown in Figure 5. The scale has a maximum size of 4 mm in width and 3.9 mm in length, with a height of 1.7 mm. Specifically, the scale is formed by a support base to the substrate, with 1.5 mm sides and a 0.4 mm height, and a shape similar to those seen in nature in fishes. This scale also has an inclination angle of 77° with respect to the vertical to ensure a geometric arrangement with overlapping and staggered scales.

**Figure 6:**
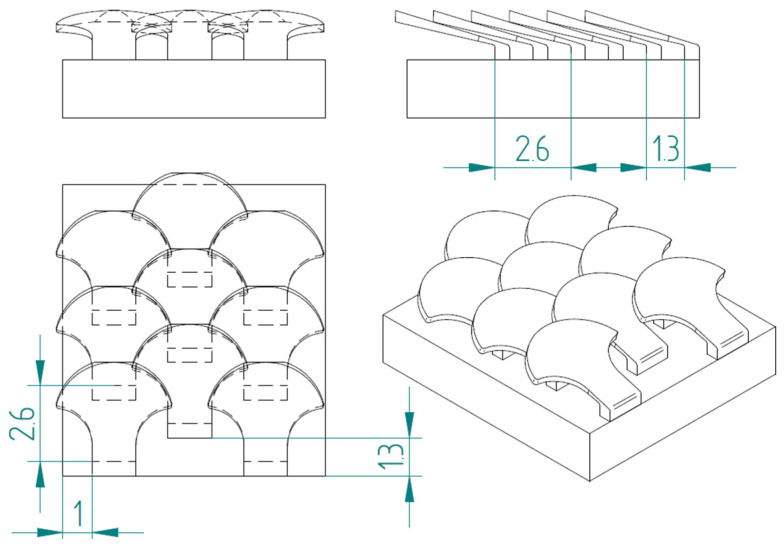
Type 1 bio-inspired scale structure in a configuration with overlapping and staggered scales.

In nature, the scales are arranged in overlapping and staggered structures, as in Figure 6, to provide greater protection to the underlying skin. This structure is made up of three staggered columns, each of which is made up of three scales. In each column, the scales are placed at a distance of 2.6 mm from each other, while the columns are offset by 1.3 mm from each other. In this way, each scale has a distance of 1.3 mm from the next, ensuring a stronger connection than the simple overlapping scale structure. In fact, the second scale of the second column, that is, the one placed in the center of the structure, is connected to the six scales that surround it, thus allowing for greater protection, thanks to the mutual actions derived from the contacts between the various scales.

#### 2.1.2. Type 2 Bio-Inspired Scale

**Figure 7:**
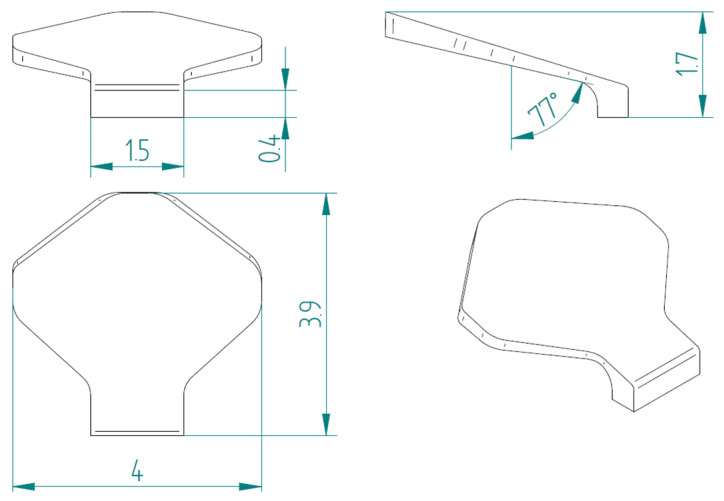
Type 2 bio-inspired scale with geometric dimensions in millimeters.

The second type, referred to as type 2, is shown in Figure 7.

**Figure 8:**
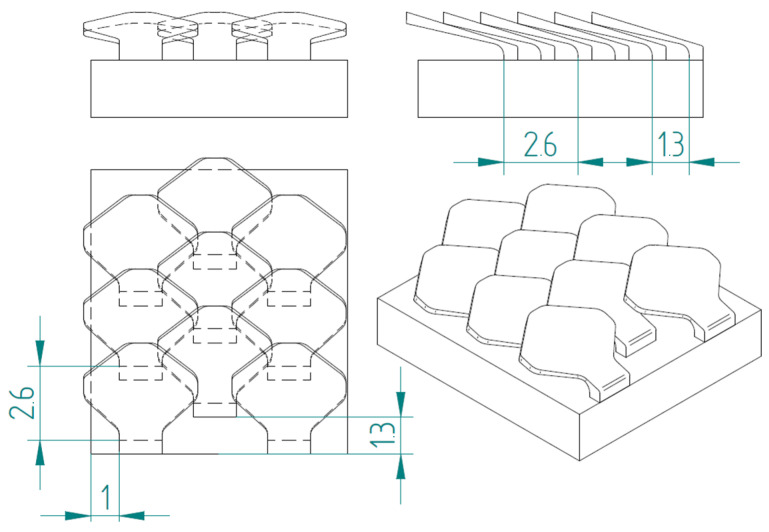
Type 2 bio-inspired scale structure in a configuration with overlapping and staggered scales.

Figure 8 shows the structure with overlapped and staggered scales for the type 2 bio-inspired scales. The scales are spaced 2.6 mm apart, while the columns are offset by 1.3 mm.

#### 2.1.3. Substrate

**Figure 9:**
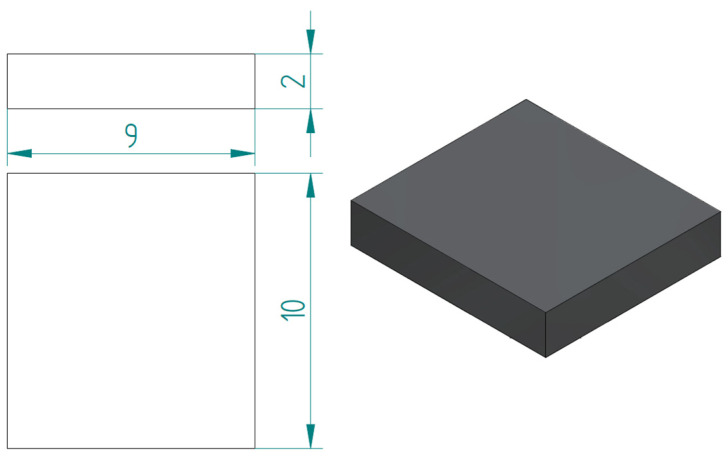
Substrate: rectangular 9 × 10 mm.

The substrate reproduces the skin of the fish, and it has been modeled in a prismatic form, which has a rectangular section with 9 mm and 10 mm sides, with a thickness of 2 mm, see Figure 9.

### 2.2. Numerical Method

Once the design of the scale structures was defined, the finite element method was used to perform numerical simulations of a puncture resistance test and a flexural strength test in order to evaluate the performance of each structural configuration with regard to the protective capacity and flexibility.

#### 2.2.1. Material Properties

As in nature, the scales must be made of a hard material resistant to impacts and perforations, while the substrate must be made of a soft and elastic material to allow for good flexibility. It is also necessary to consider that the scales could be made via 3D printing; in this perspective, it has been assumed that the scales are made of thermoplastic polymer ABS (acronitrile butadiene styrene), while the substrate is made of polyester fabric. ABS’s mechanical behavior is mainly that of a fragile material; in its stress–strain curves, the plastic behavior is almost absent. Moreover, the local stresses for the level of loads applied do not determine fracture. For this reason, for the ABS in the simulations, only the linear properties have been considered.

In particular, these properties have been considered for ABS:

- Density ρ = 1040 kg/m^3^;
- Young’s longitudinal modulus of elasticity E = 3 GPa = 3000 MPa;
- Poisson coefficient ν = 0.35.

The mechanical properties hypothesized for the polyester fabric are the following:

- Density ρ = 1200 kg/m^3^;
- Young’s longitudinal modulus of elasticity E = 0.5 GPa = 500 MPa;
- Poisson’s ratio ν = 0.4.

The puncture tests require a hard tip; it was assumed to be made of structural steel, having the following properties:

- Density ρ = 7850 kg/m^3^;
- Young’s longitudinal modulus of elasticity E = 200 GPa = 200,000 MPa;
- Poisson’s ratio ν = 0.3;
- Breaking strength σ_r_ = 460 MPa.

#### 2.2.2. Finite Element Model

The simulations were conducted using the commercial software ANSYS Workbench 2020. Nonlinear static analyses were performed with the large deflection option enabled and the number of substeps configured as follows: initial 200, minimum 100, and maximum 1000. Linear tetrahedral elements were utilized for the mesh, with element sizes assigned as follows: the scales were meshed with a body sizing of 0.15 mm and a face sizing of 0.10 mm. Additionally, in the type 1 bending simulations, the lateral faces that come into contact under high deformation levels were refined with a face sizing of 0.05 mm. The substrate was meshed with elements sized at 0.15 mm. The “Stabilization” option was left on the default: “Program controlled”.

Contact pairs are automatically created by the software when the CAD file is imported, but the properties are always set as “bonded” so it is a task of the user to change this setting where needed. For the scale–scale and scale substrate contact, a “frictional” type of contact was selected with a coefficient of friction equal to 0.2.

#### 2.2.3. Puncture Resistance Test

**Figure 10:**
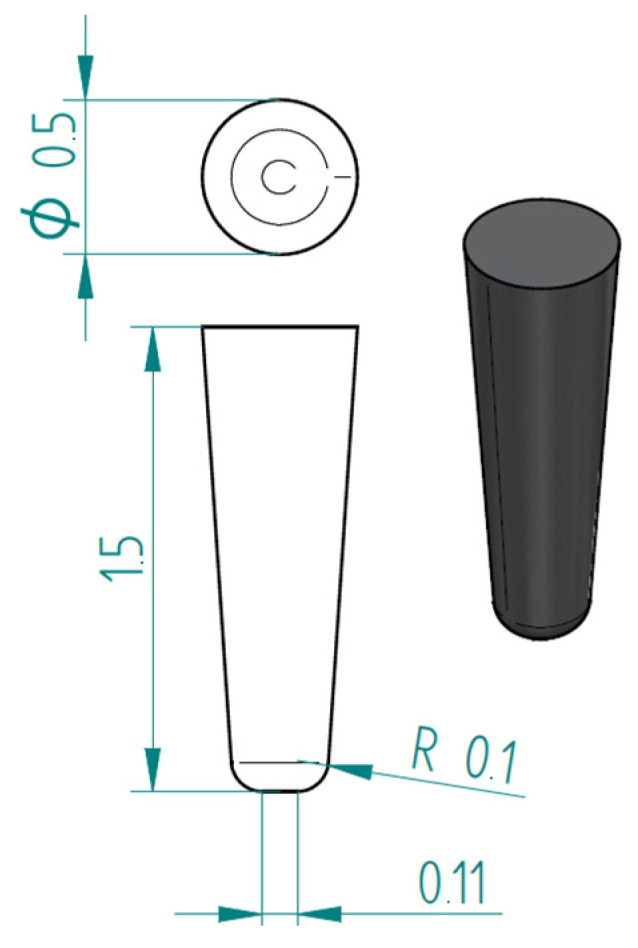
Pointed indenter that reproduces a steel needle. Dimensions are in millimeters.

The first test performed was puncture resistance; it was performed through the action of a hard tip, which causes piercing stress on the structure. The simulations reproduce the puncture tests performed by Martini et al. [4]. Figure 10 shows the modeled tip.

The test is simulated by binding the lower surface of the substrate with a joint and letting the drill bit descend 1.5 mm from the initial position, which is about 0.6 mm higher than the surface of the scale. In particular, in all tests, the tip is placed at a height of 3.5 mm from the upper edge of the substrate and is made to descend along the vertical, impeding displacements other than vertical movement. For both structures, a series of comparative tests were performed to evaluate the two types of scales.

#### 2.2.4. Flexural Test

Flexural tests were carried out on a configuration with overlapping and staggered scales, since it is believed that this is the most competitive. In the tests, since the scales on the “extrados side” do not produce any noteworthy stiffening compared to the bare polyester substrate because in this configuration the scales move away from each other, the scales were positioned on the ‘‘intrados” side of the bent samples in order to test the way in which they increase the rigidity of the sample through their interactions. The bending tests must be performed in two orthogonal directions, since the scales interact differently in the direction corresponding to the main bending direction in the animals with respect to the direction perpendicular to the latter. 

**Figure 11:**
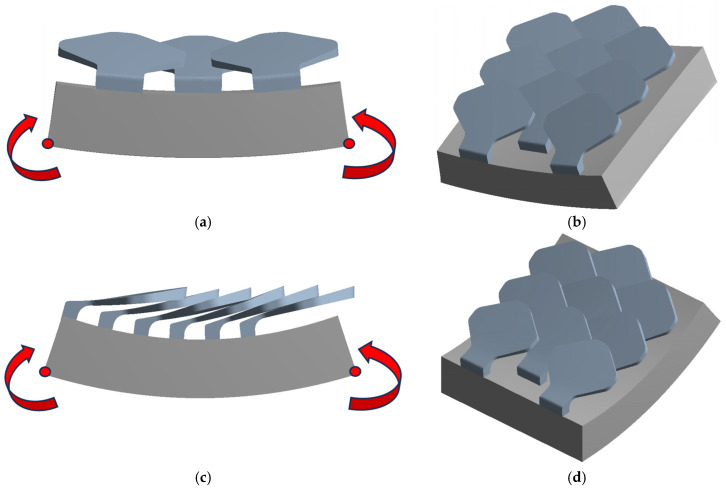
Flexural tests: (**a**) type 1—scale structure 1, (**b**) type 2—scale structure 1, (**c**) type 1—scale structure 2, (**d**) type 2—scale structure 2.

To enable high bending angles without encountering convergence issues, the boundary conditions were meticulously designed. The lateral surfaces of the substrate were connected to specific points, referred to as “remote points” in ANSYS, where rotations were applied. Additional boundary conditions were introduced solely to prevent labilities while allowing the substrate to deform freely. Refer to Figure 11 for the configurations used in the flexural tests.

## 3. Results

### 3.1. Type 1 Bio-Inspired Scale

For the overlapped and staggered scale structure, three different perforation tests were performed, in which the position of the tip was changed with respect to the perforated scale.

#### 3.1.1. Simulation Number 1: Tip at the Center of the Scale

**Figure 12:**
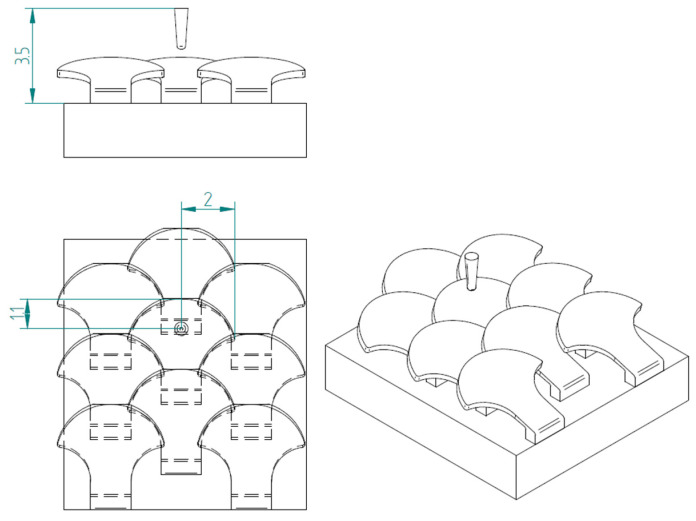
Schematic diagram of perforation—test n. 1—type 1 bio-inspired scale structure—overlapping and staggered configuration.

**Figure 13:**
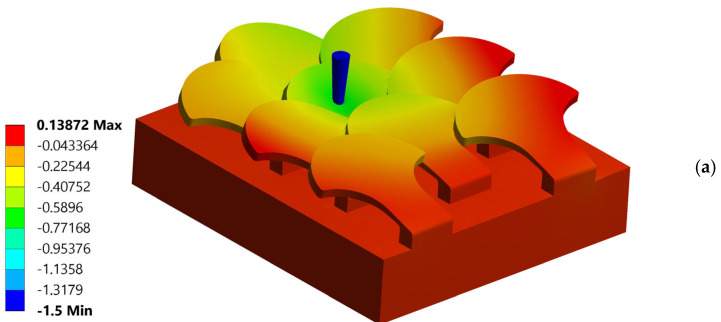

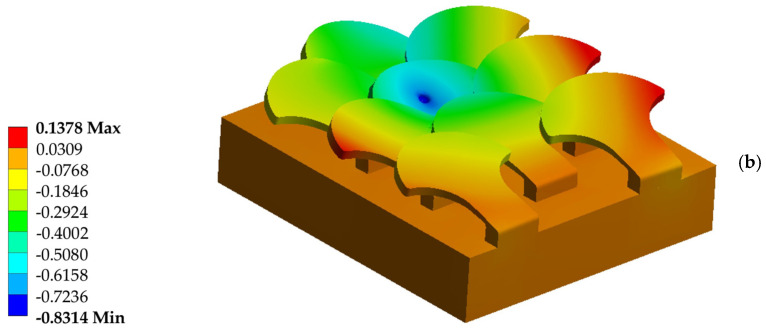
Map of displacements—puncture resistance test—test n. 1—type 1 bio-inspired scale structure—overlapping and staggered configuration: (**a**) with and (**b**) without the tip.

Figure 12 shows a schematic relating to the first simulation performed. The interconnections between the various scales in the configuration with overlapping and staggered scales support the scale where the tip deforms as a result of mutual contact but help to reduce the deformation undergone by the scale subjected to perforation. In Figure 13a,b, a map of the displacements in the z direction, respectively, with and without tip, is shown. The maximum displacement in z of the scale is 0.83 mm; see Figure 13b. The critical punching force value detected is Fp = 144 N.

#### 3.1.2. Simulation Number 2: Tip Near the Left Edge of the Scale

**Figure 14:**
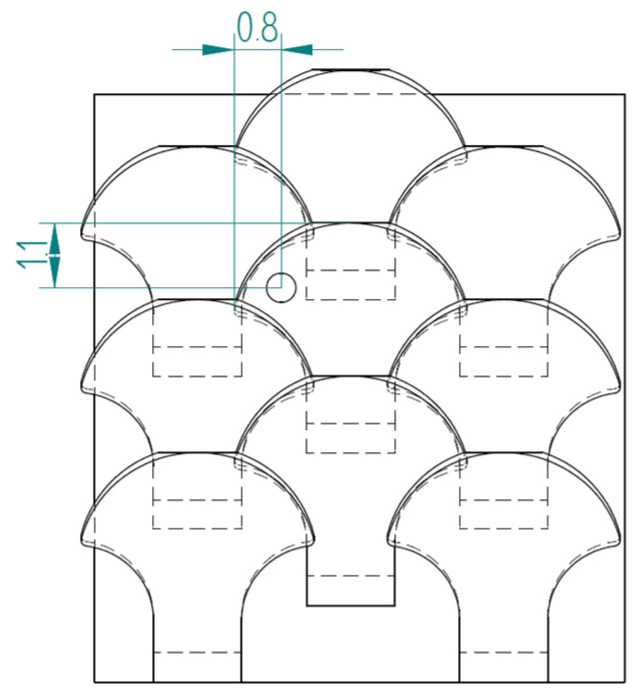
Schematic diagram of perforation test n. 2—structure of bio-inspired scales of type 1—configuration with overlapping and staggered scales.

Figure 14 shows the schematization of the subsequent perforation test performed, in which the tip acts near the left edge of the previously perforated scale. In particular, the tip is positioned at a distance of 1.1 mm from the upper edge of the scale, as in the previous case, and at a distance of 0.8 mm from the left end of the scale.

**Figure 15:**
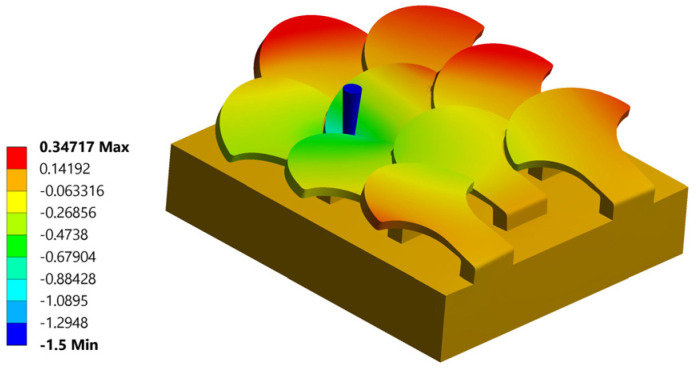
Perforation test n. 2 bio-inspired structure 1—overlapping and staggered scales: total displacements in mm.

Figure 15 shows that the scale tends to tilt to the left due to the action of the tip placed there. In this case, the maximum displacement in z of the scale is 0.85 mm, slightly higher than seen in the previous case. Overall, there is a greater deformation of the scale. In fact, the critical drilling force value detected is Fp = 56 N, much lower than the value obtained if the tip acts symmetrically with respect to the scale.

#### 3.1.3. Simulation Number 3: Tip Near the Upper Edge of the Scale

**Figure 16:**
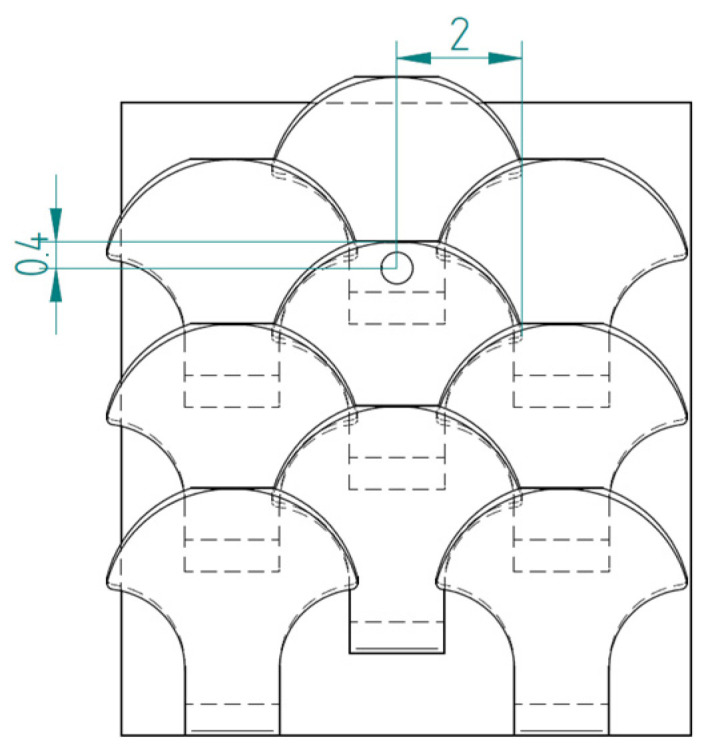
Schematic diagram of perforation test n. 3—type 1 bio-inspired scale structure—configuration with overlapping and staggered scales.

The last simulation performed is schematized in Figure 16. In the last test, the tip acts symmetrically with respect to the scale, but near the upper edge, i.e., at a distance of 0.4 mm, as shown in Figure 16.

**Figure 17:**
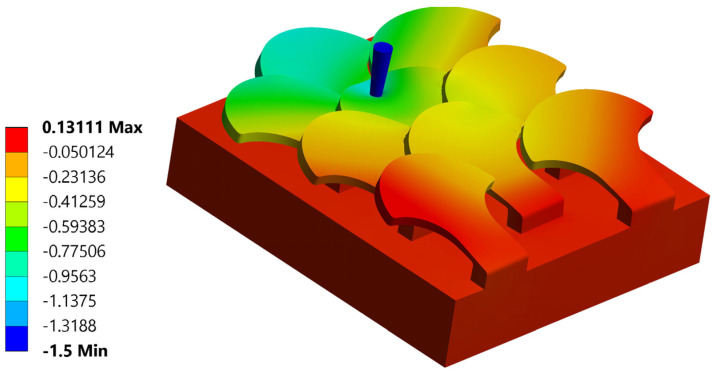
Perforation test n. 3 bio-inspired structure 1—overlapping and staggered scales: total displacements in mm.

As shown by Figure 17, the action of the tip this time causes significant deformations towards the scale. In fact, the maximum displacement in z increases considerably compared to the previous cases, reaching 1.05 mm in the area of the tip impression. To deform the scale, however, the tip must apply greater force than test number 2, in which the tip acted near the left edge of the scale. In fact, the critical force value detected is Fp = 136 N, greater than the value found for simulation number 2. This is due to the fact that, in the latter case, the tip acts symmetrically with respect to the scale, favoring contacts between the nearby scales, which increase the protection of the structure. As noted, the critical drilling force of this configuration is lower than that found in test number 1, due to the fact that the tip is located closer to the edge of the scale, causing a greater bending load on it.

### 3.2. Type 2 Bio-Inspired Scale Structure

The simulations performed for the type 2 bio-inspired scale structure are the same as those performed for the type 1 bio-inspired structure. These are detailed in the following paragraphs.

#### 3.2.1. Simulation Number 1: Tip Near the Center of the Scale

**Figure 18:**
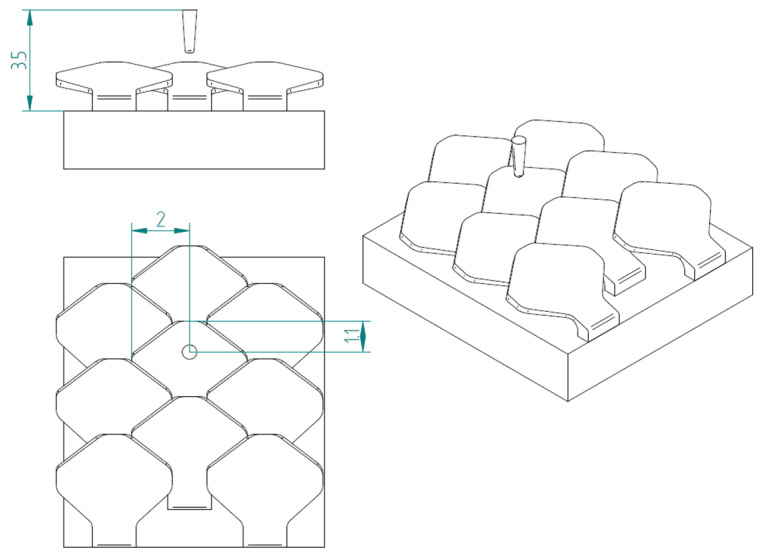
Schematic diagram of perforation test n. 1—type 2 bio-inspired scale structure—configuration with overlapping and staggered scales.

The first analysis is schematized in Figure 18. The tip is placed in the same position as seen for the type 1 bio-inspired structure.

**Figure 19:**
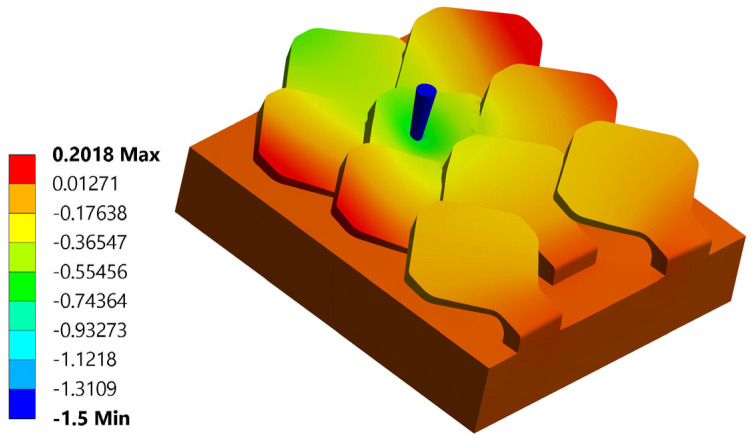
Perforation test n. 1 bio-inspired structure 2—overlapping and staggered scales: total displacements in mm.

Analyzing the displacement in z of the single perforated scale, the same behavior seen with type 1 bio-inspired scales is recorded. In this regard, Figure 19 shows the displacement along z. The maximum displacement suffered by the scale is 0.83 mm, in accordance with the value found for the type 1 scale structure, but for this configuration, there is a reduction in the overall deformation suffered by the bio-inspired type 2 scale compared to that seen for the type 1 scale in the same load condition. This aspect is certainly linked to the geometric shape of the type 2 scale, which ensures better interconnection between the various scales. For this load condition, the critical piercing force is Fp = 156 N. The critical piercing force value detected for this configuration is greater than the critical piercing force evaluated for the same load configuration with the type 1 scale structure.

#### 3.2.2. Simulation Number 2: Tip Near the Left Edge of the Scale

**Figure 20:**
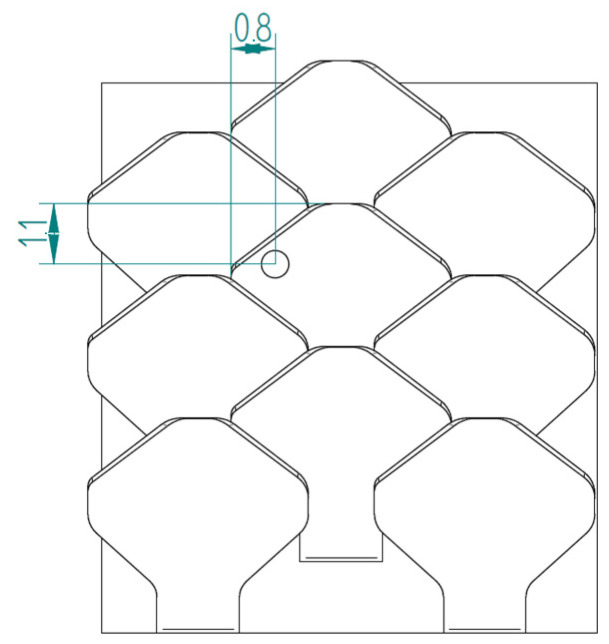
Schematic diagram of perforation test n. 2—type 2 bio-inspired scale structure—configuration with overlapping and staggered scales.

In this case, the tip is placed near the left edge of the scale; see Figure 20.

**Figure 21:**
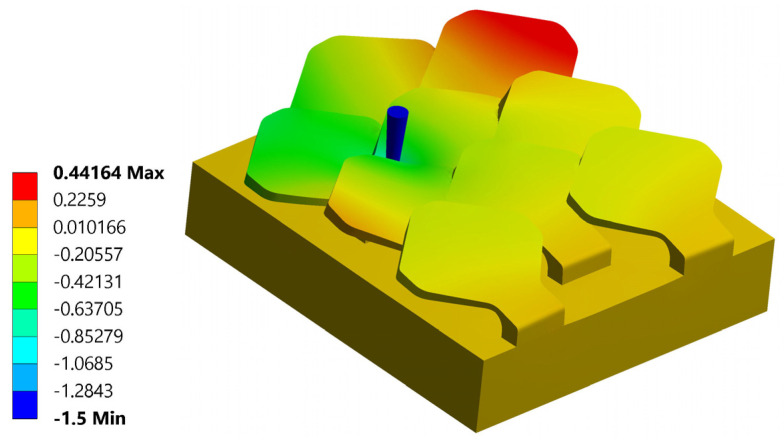
Perforation test n. 2 bio-inspired structure 2—overlapping and staggered scales: total displacements in mm.

Figure 21 shows the final deformed configuration. This detail highlights the asymmetric deformation suffered by the scale. Furthermore, the maximum displacement value in z suffered by the scale in the area of the tip is 0.83 mm. The latter is equal to the displacement in z suffered by the scale during simulation number 1, in which the tip acted near the center of the scale. This result shows that the structure with overlapping and staggered scales with type 2 bio-inspired scales is very efficient even in the presence of a perforating load that acts asymmetrically against the scale. The improved efficiency of the bio-inspired type 2 scale is highlighted once again by the detected critical piercing force, which is equal to Fp = 82 N. This value is much higher than that found for the type 1 bio-inspired scale structure with the same load conditions.

#### 3.2.3. Simulation Number 3: Tip Near the Upper Edge of the Scale

**Figure 22:**
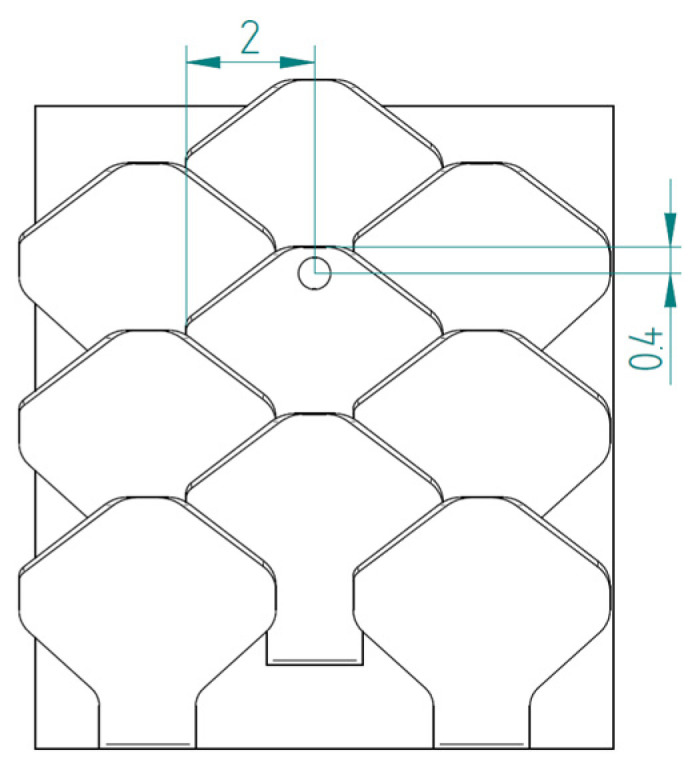
Schematic diagram of perforation test n. 3—type 2 bio-inspired scale structure—configuration with overlapping and staggered scales.

**Figure 23:**
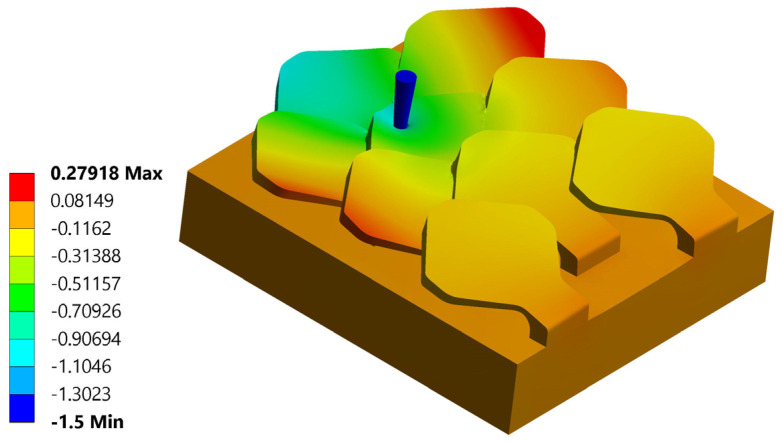
Perforation test n. 3 bio-inspired structure 2—overlapping and staggered scales: total displacements in mm.

The last perforation test performed is schematized in Figure 22. Below, in Figure 23, the map of the total displacements is illustrated. In this load condition, the tip causes significant deflections to the affected scale, thus generating significant deformations also to the neighboring scales. From the images below, you can appreciate the symmetrical behavior of the structure following the application of the load.

This best illustrates the significant deflections suffered by the scale. In fact, the maximum displacement detected in the load application area is 1.03 mm. This value is comparable with the outcomes of simulation n. 3 performed for the type 1 structures. The interconnections between the neighboring scales, however, cause the structure to offer higher resistance to these deflections. In fact, the critical drilling force value is Fp = 158 N, the highest recorded in all the eight tests performed for overlapping and staggered scale structures.

### 3.3. Comparison Between the Two Bio-Inspired Scales

It emerged that the bio-inspired type 2 scale is more resistant to perforation than the bio-inspired type 1 scale.

To have a quantitative comparison between the two types of scales, we decided to use the perforation response surfaces as the point of action of the perforating tip changes, similarly to what was carried out by R. Martini, Y. Balit, and F. Barthelat [4].

To do this, it was necessary to perform six additional perforation tests compared to the three tests previously performed for the overlapped and staggered structures with type 1 and type 2 scales.

**Figure 24:**
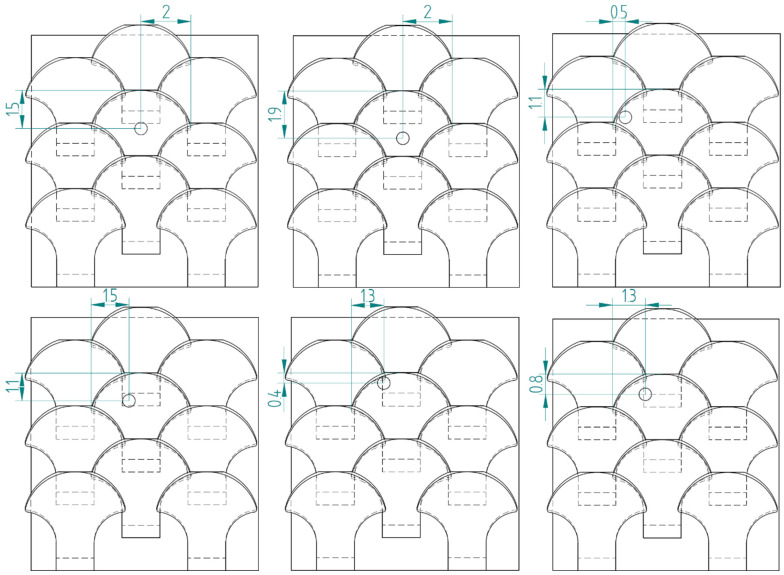
Schematization of six additional perforation tests—type 1 bio-inspired scale structure—overlapping and staggered scale configuration.

Figure 24 shows the schematizations of the six additional tests performed for a type 1 bio-inspired scale structure.

**Figure 25:**
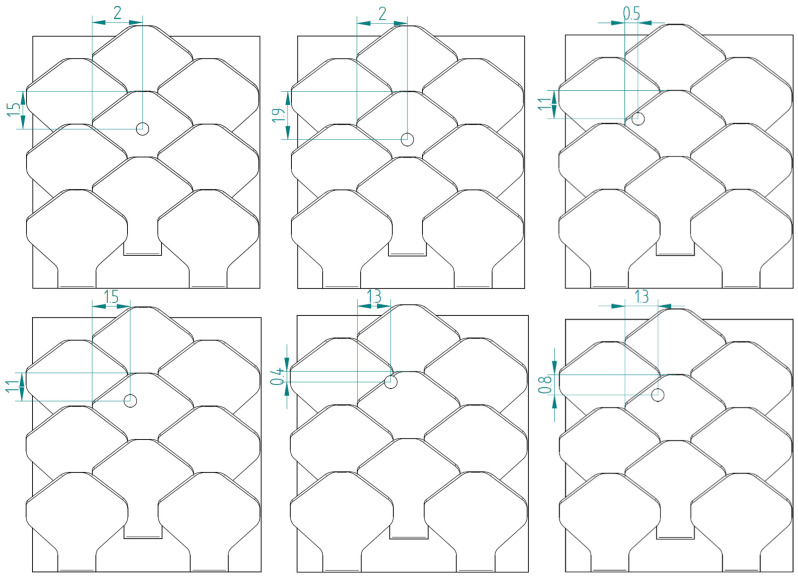
Schematization of six additional perforation tests—type 2 bio-inspired scale structure—overlapping and staggered scale configuration.

Figure 25, on the other hand, shows the schematization of the six additional tests carried out for the bio-inspired type 2 scale structure. The tests performed are the same as those carried out for the type 1 scale structure, in order to carry out a direct comparison among the data found.

In the various analyses performed, the tip varies with respect to the axis of symmetry of the perforated scale, with any offsets only towards the left part of the scale. This is carried out because the same results are obtained if the tip is positioned specularly with respect to the axis of symmetry of the scale. Below, in Table 1, the critical penetrating force values obtained in the various analyses performed for the structure with bio-inspired stairs of type 1 and type 2 are shown. Table 1 reports both the results of the new group of analyses 4, 5, 6, 7, 8, and 9 and the results of analyses 1, 2, and 3 previously illustrated in the appropriate sections.

**Figure 26:**
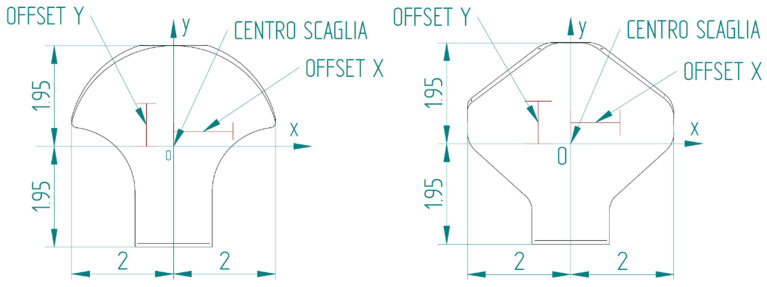
x-y coordinate system used to report the position where the tip hits the scale. Type 1 bio-inspired scale on the left; type 2 bio-inspired scale on the right.

The position where the tip strikes the scale is also included in the table. This position is evaluated with offsets in the x and y directions with respect to the center of the scale, as shown in Figure 26.

Table 1 allows for two interesting considerations:

- Very low puncture resistance values were found in analysis number 5, in which the tip is placed near the center of the scale. This is due to the fact that at that point, the affected scale is more exposed, since it is less helped by the nearby scales, due to the degree of overlap used;
- Analysis number 9 is the only one in which the type 1 bio-inspired scale structure has a greater perforation resistance than the type 2 bio-inspired scale structure.

**Figure 27:**
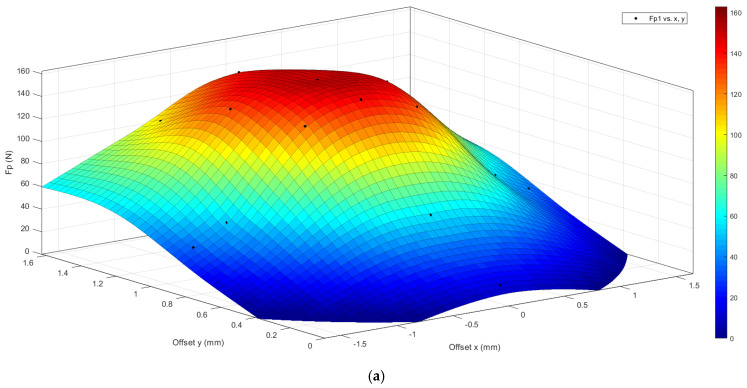

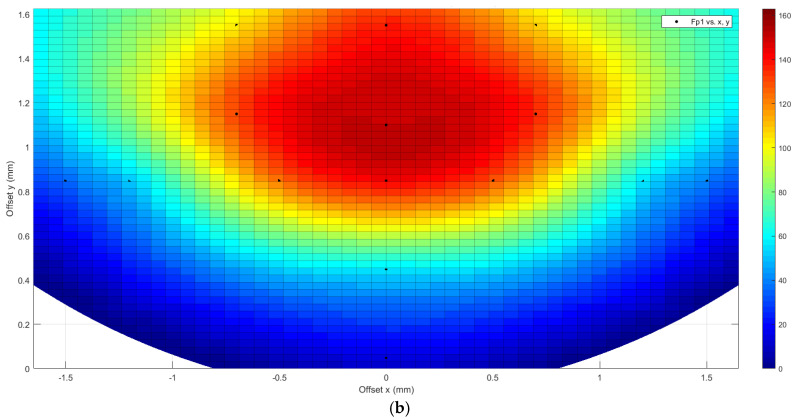
Map of critical drilling force as the action point of the drill bit changes (offset in x and in y)—type 1 bio-inspired scale structure—high response surface, low grid of force values in x and y (the colored bar indicates the entity of the punching force detected: dark red, max values; blue, minimum values).

Having performed nine different perforation simulations, it is possible to reconstruct the trend of the critical perforation force as the point where the scale is hit. In Figure 27a,b, the trend of the critical drilling force as the offset changes in x and y is shown with respect to the center of the scale for a type 1 bio-inspired scale structure.

**Figure 28:**
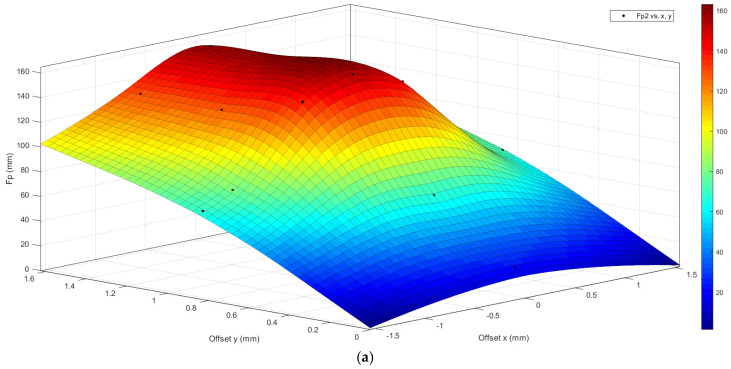

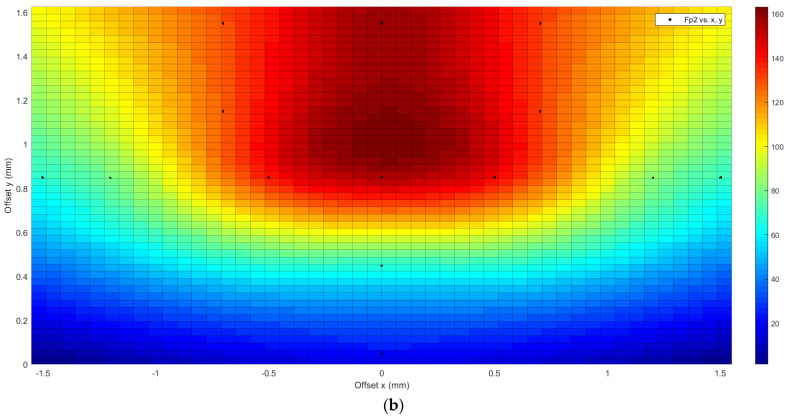
Map of critical drilling force as the action point of the drill bit changes (offset in x and in y)—type 2 bio-inspired scale structure—high response surface, low grid of strength values in x and y (the legend indicates the entity of the punching force detected: dark red, max values; blue, minimum values).

Figure 28 shows the same graphs for a type 2 bio-inspirate scale structure.

The graphs shown are constructed by interpolation starting from the results obtained in the nine analyses shown in Table 1. The graphs once again highlight the greater puncture resistance of the type 2 bio-inspired scale structure compared to type 1.

**Figure 29:**
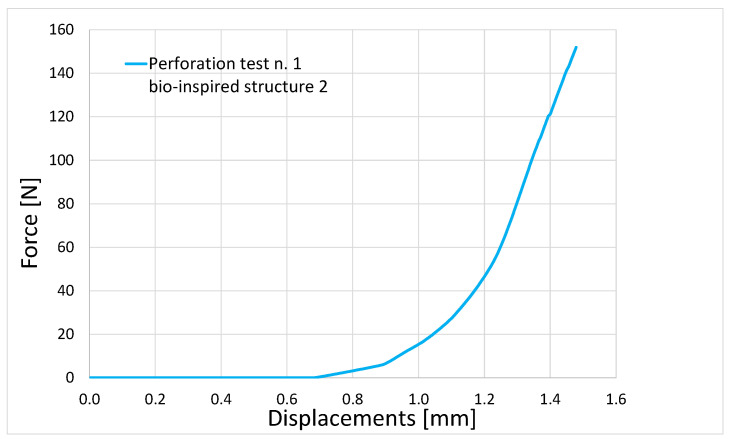
Force as a function of the tip displacement for the case where the highest reaction force was recorded: perforation test n. 1 of bio-inspired structure 2.

Figure 29 presents an example of the force as a function of the tip displacement for the case where the highest reaction force was recorded, specifically during perforation test n. 1 of bio-inspired structure 2.

### 3.4. Flexural Test

#### 3.4.1. Type 1 Bio-Inspired Scale Structure

**Figure 30:**
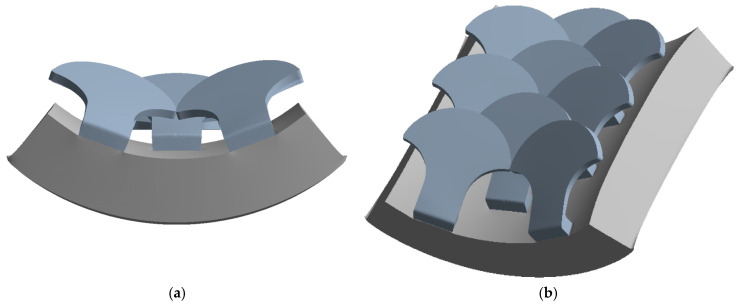
Flexural test—type 1—bio-inspired structure 1.

Figure 30a,b show the final deformed configuration for bending test n. 1 for the type 1 bio-inspired scale structure. Once again, it is necessary to define a parameter that characterizes the performance of the structure following the load applied. As described in Section 2.2.4, the lateral surfaces of the substrate were connected to specific points, referred to as “remote points” in ANSYS, where rotations were applied to influence the connected surfaces. Additional boundary conditions were introduced solely to prevent instability while allowing the substrate to deform freely. Both lateral surfaces were rotated by 40 degrees. In Flexural Test 1, the total bending moment, calculated as the sum of the moments at the two distinct remote points, is M_1_ = 1023 Nmm.

**Figure 31:**
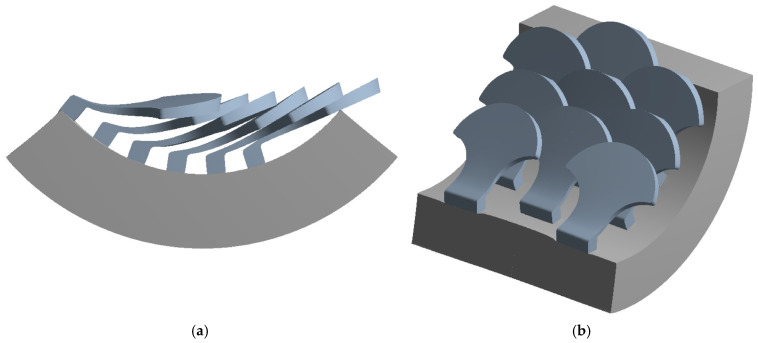
Flexural test—type 2—bio-inspired structure 1.

The second bending test is illustrated in Figure 31 below, which shows the final deformed configuration at the end of the analysis. The bending of the substrate is partially prevented by the scales, which come into contact with the substrate itself. The bending moment detected for the second analysis is M_2_ = 1505 Nmm.

#### 3.4.2. Type 2 Bio-Inspired Scale Structure

**Figure 32:**
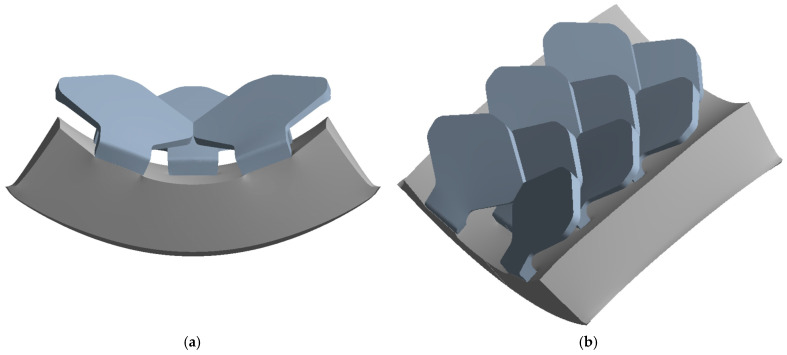
Flexural test—type 1—bio-inspired structure 2: (**a**) Front view, (**b**) Isometric view.

The same tests seen in the previous paragraph are repeated for the type 2 bio-inspired scale structure. Figure 32 shows the total deformed configuration from bending test n. 1.

The measured bending moment is M_1_ = 1092 Nmm. This value exceeded the value of 1023 Nmm observed in the bending analysis of the test conducted for the type 1 scale structure.

**Figure 33:**
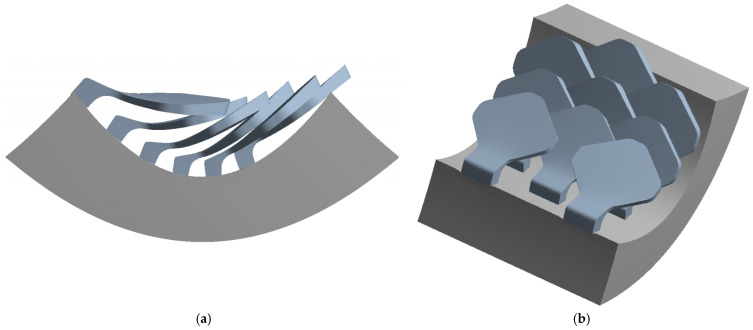
Flexural test—type 2—bio-inspired structure 2: (**a**) Front view, (**b**) Isometric view.

Finally, Figure 33 shows the deformed structure from bending test number 2.

The bending moment detected is M_2_ = 1574 Nmm, greater than the value of 1505 Nmm found for bending analysis number 2 performed for the type 1 scale structure. 

## 4. Discussion

A small variation in the geometry of the scale can greatly influence the behavior of the structure in terms of puncture resistance and flexibility. This can be seen in the comparison between the type 1 bio-inspired scale and the type 2 scale. These two scales, the same in material, are similar geometrically: they differ from each other in small features, while maintaining the same overall dimensions. However, we have found that the bio-inspired type 2 scale is more efficient in terms of puncture resistance than the bio-inspired type 1 scale, while the latter guarantees slightly better flexural behavior.

In puncture resistance tests, the type 2 scale outperforms the type 1 scale primarily due to its shape, which results in a greater degree of overlap with the surrounding scales, and partially from its shape. Since the distribution of the scales on the substrate is identical for both types, it does not influence the comparison.

In the flexural test for type 1, the sliding of the two outer rows of scales over the central row initially generated similar friction forces in both cases, as the differing shapes of the scales had minimal impact on friction, until higher bending angles were reached. At these higher angles, the outer rows of scales come into contact along their thickness. Due to the distinct shapes of the two scale types, the forces exchanged during this contact differ.

For the flexural test of type 2, at high bending angles, all the scales undergo significant bending. Since the bending stiffness varies between the two scale types due to their unique shapes, the results diverge.

Furthermore, the performance of the scale structure is strongly influenced by the distance between the various scales, as well as by their arrangement. The closer they are to each other, the greater the puncture resistance, but at the same time, flexibility is reduced and weight increases.

Depending on the intended application of the protective system, you can choose a design that prioritizes greater puncture resistance or one that is lighter and more flexible.

## 5. Conclusions

The purpose of this work was to continue studies on the creation of protective structures inspired by fish scales. These can be designed for various purposes, such as bulletproof vests, protective gloves, fireproof systems, or others.

Two bio-inspired protective systems were designed and tested. Specifically, two types of scales were modeled using 3D CAD software SolidEdge 2020. Then, a configuration with overlapping and staggered scales was defined.

The proposed novel scale design differs from traditional flat artificial scales by incorporating a vertical support that transitions into an inclined protective surface. The design aims to increase the distance between the top of the scale and the substrate, requiring greater deformation of the scales when subjected to pressure. This enhanced deformation allows the scales to absorb more energy, thereby improving the substrate’s ability to withstand loads. In contrast, the traditional flat scale design merely provides a hard surface to shield the softer substrate and redistributes pressure over a larger area. The scales are meticulously spaced to enable slight overlapping at the edges, offering effective protection while maintaining the structure’s lightness and flexibility. Their thickness is thoughtfully chosen to provide the strength necessary to withstand loads through deformation, without compromising their lightweight nature.

The two bio-inspired structures were analyzed by finite element performance tests. Specifically, the structures were tested for their guaranteed protection using perforation tests and for their flexibility using flexural tests. The execution of these simulations allowed for a complete comparative analysis between the two types of scales. The analyses carried out showed that the type 2 scale is more efficient in terms of puncture resistance, while the type 1 scale has better performance in terms of flexibility.

The small differences in the two scales demonstrate that small geometric variations can significantly influence the behavior of the protective system, leaning towards greater protection or towards greater flexibility. These two aspects, in fact, influence each other and, depending on the use to be made of the protective system in question, you can opt for a solution that guarantees greater protection at the expense of flexibility or greater capacity of movement at the expense of the guaranteed protection.

The models presented and analyzed can be considered as a starting point for subsequent analyses or modeling of structures that have the purpose of imitating the protective systems present in nature. To improve and optimize the protective systems, you can act in different ways, varying the following:

- Scale material;
- Substrate construction material;
- Geometric structure of the scale: dimensions, shape, and thickness;
- Distance between the various scales placed above the substrate;
- Types of reciprocal dispositions of the scales: with scales placed side by side, with overlapping scales, and with overlapping and staggered scales;
- The ratio between the surface area of the scale and the distance between their centers;
- The ratio between the area of the connection to the substrate and the area of the scale.

In the future, we can envision developing protective systems entirely inspired by nature, striving, if possible, to surpass the capabilities currently found in natural designs.

### Future Work

Most studies on impact and penetration issues involving bio-inspired protective scales focus primarily on the behavior of individual scales. However, in many scenarios, multiple scales are subjected to extreme loading simultaneously. Future research should explore this context, particularly the complex interactions between scales under such conditions, offering a fascinating and largely uncharted avenue for investigation.

## Figures and Tables

**Table 1:** Values of critical drilling force Fp with variation in the penetrator action point.

Number of Analyses	Point of Action of the Tip with Respect to the Center of the Scale		Critical Drilling Force Fp (N)	
	Offset X (mm)	Offset Y (mm)	Structure Bio 1	Structure Bio 2
1	0	0.85	144 N	156 N
2	−1.2	0.85	56 N	82 N
3	0	1.55	136 N	158 N
4	0	0.45	60 N	70 N
5	0	0.05	16 N	23 N
6	−1.5	0.85	39 N	70 N
7	−0.5	0.85	129 N	142 N
8	−0.7	1.55	105 N	131 N
9	−0.7	1.15	134 N	130 N

## Data Availability

The data presented in this study are available from the corresponding author upon request.
